# Treatment de-escalation in Philadelphia chromosome–positive B-cell acute lymphoblastic leukemia: the emerging role of chemotherapy-free regimens

**DOI:** 10.1177/20406207231151294

**Published:** 2023-02-03

**Authors:** Fadi G. Haddad, Jacki Sawyers, Nicholas J. Short

**Affiliations:** Department of Leukemia, The University of Texas MD Anderson Cancer Center, Houston, TX, USA; Department of Leukemia, The University of Texas MD Anderson Cancer Center, Houston, TX, USA; Department of Leukemia, The University of Texas MD Anderson Cancer Center, 1515 Holcombe Blvd. Unit 428, Houston, TX 77030, USA

**Keywords:** BCR::ABL1, blinatumomab, chemotherapy, ponatinib, tyrosine kinase inhibitor

## Abstract

The management of Philadelphia chromosome-positive (Ph-positive) acute lymphoblastic leukemia (ALL) has witnessed major progress over the past two decades. Initially, the incorporation of the first-generation BCR::ABL1 tyrosine kinase inhibitor (TKI) imatinib into intensive chemotherapy regimens improved outcomes compared with chemotherapy alone. The combinations of chemotherapy with second- or third-generation TKIs further improved outcomes, with higher rates of complete molecular remission (CMR) and superior survival. The combination of ponatinib plus chemotherapy resulted in durable remissions and prolonged long-term survival, even in patients who did not receive allogeneic stem cell transplantation (SCT). The promising results seen with later-generation TKIs have caused many to re-evaluate the role of allogeneic SCT for patients who achieve CMR with potent TKI regimens. Recently, the chemotherapy-free combinations of blinatumomab plus TKIs were shown to be safe and effective in newly diagnosed Ph-positive ALL, sparing patients the toxicities associated with intensive chemotherapy. In particular, encouraging early results have been seen with the combination of blinatumomab plus ponatinib, suggesting that this regimen may represent a chemotherapy-free and SCT-sparing strategy for patients with Ph-positive ALL. Herein, we discuss the current evidence for frontline therapies of Ph-positive ALL, the treatment de-escalation strategies over time, and the role of allogeneic SCT in view of the emergence of newer chemotherapy-free regimens using potent TKIs.

## Introduction

Philadelphia chromosome (Ph)-positive acute lymphoblastic leukemia (ALL) was historically associated with a very poor prognosis. Treatment relied on induction chemotherapy followed by allogeneic stem cell transplantation (SCT) in first complete remission (CR), with long-term survival rates of around 10–35%.^1^ The addition of BCR::ABL1 tyrosine kinase inhibitors (TKIs) to backbone chemotherapy has significantly improved outcomes. Four different BCR::ABL1 TKIs have been combined with chemotherapy in prospective studies of patients with Ph-positive ALL: the first-generation TKI imatinib, the second-generation TKIs dasatinib and nilotinib, and the third-generation TKI ponatinib. The incorporation of newer and more potent TKIs into chemotherapy regimens has increased the rates of complete molecular remission [CMR; generally defined as undetectable *BCR::ABL1* transcripts by polymerase chain reaction (PCR)], which, in turn, improved long-term survival from approximately 40% with imatinib to 75% with ponatinib, with less reliance on allogeneic SCT when later-generation TKIs are used.^2^

The CD3/CD19 bispecific T-cell engaging antibody blinatumomab improves the outcomes of patients with relapsed/refractory B-cell ALL, compared with standard chemotherapy and is also active in Ph-positive ALL.^3,4^ The combination of blinatumomab with TKIs demonstrated high clinical activity in retrospective studies of patients with relapsed/refractory Ph-positive ALL.^5–7^ More recently, clinical trials have been conducted to evaluate the role of blinatumomab in combination with TKIs in patients with newly diagnosed Ph-positive ALL. Herein, we discuss treatment de-escalation in Ph-positive ALL from intensive chemotherapy combinations to chemotherapy-free regimens and highlight the promise of novel blinatumomab plus TKI regimens for this disease, which are transforming Ph-positive ALL from one of the most aggressive and deadly forms of leukemia to now one of the most curable.

## Literature search

During the preparation of this review, we searched PubMed for studies on the outcomes of adults with Ph-positive ALL and clinical trials in this population without date restriction. We used the following keywords for the search: ‘acute lymphoblastic leukemia’, ‘Philadelphia chromosome-positive’, ‘tyrosine kinase inhibitor’, ‘imatinib’, ‘dasatinib’, ‘nilotinib’, ‘ponatinib’, and ‘blinatumomab’. We selected the most relevant studies to be included in this article for each TKI and according to the intensity of therapy.

## Combinations of TKIs with intensive chemotherapy

### First-generation TKI: imatinib

Imatinib was the first BCR::ABL1 TKI to be used in patients with Ph-positive ALL, initially alone, and then in combination with intensive chemotherapy.^8,9^ The combination of imatinib with Hyper-CVAD (hyperfractionated cyclophosphamide, vincristine, doxorubicin, and dexamethasone, alternating with high-dose methotrexate and cytarabine) was evaluated in 54 patients (median age = 51 years) with newly diagnosed or minimally treated Ph-positive ALL.^8^ The CR rate was 93% and 16 (30%) patients underwent allogeneic SCT in first remission, which translated into a 5-year overall survival (OS) of 43%. These outcomes were corroborated in the large ECOG 2993/UKALLXII study also combining imatinib with intensive chemotherapy.^10^ Results from this study showed that, compared with the pre-imatinib era, the addition of imatinib induced higher CR rates (92% *versus* 82%; *p* = 0.004) and allowed more patients to transition to an allogeneic SCT (46% *versus* 31%), which resulted in superior OS (4-year OS: 38% *versus* 22%; *p* = 0.003). These findings showed for the first time that the addition of TKIs to chemotherapy improved the outcome of patients with Ph-positive ALL and facilitated bridging to a potentially curative allogeneic SCT.

### Second-generation TKIs: dasatinib and nilotinib

Further improvement in the outcomes of patients with Ph-positive ALL was seen with the introduction of second-generation TKIs, dasatinib and nilotinib.^11–13^ Dasatinib was evaluated in combination with Hyper-CVAD in a phase II study of 72 patients (median age = 55 years) with newly diagnosed Ph-positive ALL.^14^ The rates of CR and CMR were 96% and 65%, respectively, with a median OS of 47 months and an estimated 5-year OS of 46%. Only 12 (17%) patients underwent allogeneic SCT in first remission. In a confirmatory trial by the Southwest Oncology Group, 97 younger patients (median age = 44 years) with Ph-positive ALL were treated with Hyper-CVAD plus dasatinib in the frontline setting.^15^ All patients who achieved remission were offered consolidation with an allogeneic SCT. The rate of CR or CR with incomplete count recovery (CRi) was 88%, and 41 patients (42%) underwent allogeneic SCT. The 3-year rates of OS and relapse-free survival (RFS) were 69% and 62%, respectively. In a landmark analysis, allogeneic SCT was associated with statistically superior RFS (*p* = 0.038) and OS (*p* = 0.037).^15^ No measurable residual disease (MRD) information, however, has been reported from this study, so it is not clear whether the benefit of allogeneic SCT might be limited to those with suboptimal MRD responses.

Similar outcomes were observed in a recent study from Japan of 78 patients (median age = 45 years) with Ph-positive ALL treated with a dasatinib-based two-step induction: induction 1 with dasatinib plus prednisolone, followed by induction 2 with intensive chemotherapy and dasatinib.^16^ All patients achieved CR or CRi after induction 1, and 58% of patients achieved MRD negativity by real-time quantitative PCR after the first cycle of consolidation. Fifty-eight (74%) patients underwent allogeneic SCT in first remission. At a median follow-up for 4 years, 3-year event-free survival (EFS) and OS rates were 66% and 81%, respectively.

Intensive chemotherapy was also combined with nilotinib in 30 patients (median age = 40 years) and resulted in a cumulative CMR rate of 83%.^17^ Overall, 53% of patients underwent allogeneic SCT in first remission, and the 4-year OS rate was 45%.

In the absence of randomized studies comparing BCR::ABL1 TKIs in adults with Ph-positive ALL, a recent study in a pediatric population of Ph-positive ALL provides some guidance.^18^ In this phase III trial, 189 patients (up to the age of 18 years) were randomized to receive chemotherapy with imatinib 300 mg/m^2^/day or dasatinib 80 mg/m^2^/day, a dose of dasatinib associated with enhanced central nervous system (CNS) penetration.^19^ Dasatinib resulted in higher rates of 4-year EFS (71% *versus* 48.9%; *p* = 0.005) and 4-year OS (88.4% *versus* 69.2%; *p* = 0.04) compared with imatinib. Dasatinib was also associated with a lower cumulative risk of relapse (4-year rate of relapse, 19.8% *versus* 34.4%; *p* = 0.01), which was driven, in part, by a lower risk of isolated CNS relapses (4-year rate, 2.7% *versus* 8.4%; *p* = 0.06).^18^ In combination with data with single-arm studies, this randomized study provides strong evidence for the ability of second-generation TKI to induce higher rates of molecular remission and superior survival, as compared with first-generation TKI regimens.

### Third-generation TKI: ponatinib

Despite the improvement in long-term survival to around 40–65% with the introduction of first- and second-generation TKIs, relapses still occur, mostly among patients who do not receive consolidative allogeneic SCT. Two major factors drive relapses in this setting: (1) the emergence of T315I mutations in *ABL1* detected in up to 75% of patients at the time of relapse^20^ and (2) a relatively low CMR rate (around 20% with low-intensity combinations and 50% with high-intensity combinations using first- or second-generation TKIs).

Ponatinib is a pan-BCR::ABL1 TKI that is active against the T315I resistance *ABL1* mutation. In a phase II study, 86 patients (median age = 46 years) with newly diagnosed Ph-positive ALL received Hyper-CVAD plus ponatinib.^21,22^ All patients achieved CR and 84% achieved CMR, with estimated 5-year EFS and OS rates of 68% and 73%, respectively. Only 19 (22%) patients underwent an allogeneic SCT in first remission. In a 6-month landmark analysis, there was a trend toward a better survival in nontransplanted patients (5-year OS = 86% *versus* 69%; *p* = 0.08); however, patients who proceeded to an allogeneic SCT were generally higher risk (e.g. had persistently detectable *BCR::ABL1* transcripts).^23^ These findings suggest that the vast majority of patients treated with Hyper-CVAD plus ponatinib achieve CMR, and these patients have excellent long-term survival and without the routine use of allogeneic SCT. The superiority of ponatinib compared with a second-generation TKI was suggested by a propensity-score matched analysis in which the combination of Hyper-CVAD plus ponatinib induced higher 3-month CMR rates (84% *versus* 63%; *p* = 0.25) and 3-year OS rates (83% *versus* 56%; *p* = 0.03) compared with Hyper-CVAD plus dasatinib.^24^

## Low-intensity therapies

### Combinations of TKIs with low-dose chemotherapy or corticosteroids

The incidence of Ph-positive ALL increases with age, and therefore, many older or unfit patients are not candidates for intensive chemotherapy due to the high risk of morbidity and mortality. Different combinations strategies have been designed in this setting (Table 1). Dasatinib was combined with low-intensity chemotherapy in 71 older patients with a median age of 69 years (range = 59–83 years). The rates of CR and major molecular response (MMR; ⩽3-log reduction in *BCR::ABL1* transcript levels) were 96% and 65%, respectively, and only 7 (10%) patients underwent allogeneic SCT, resulting in 5-year OS of 36%.^20^ Nilotinib was also combined with low-intensity chemotherapy in 72 older patients with a median age of 66 years (range = 55–85 years). The rates of CR and CMR were 94% and 58%, respectively. Allogeneic SCT was performed in 24 (33%) patients and resulted in lower rates of relapses (cumulative incidence of relapse: 32% in transplanted *versus* 47% in nontransplanted patients). The 4-year EFS was 42%, and the 4-year OS was 47%.^25^ Ponatinib at the dose of 45 mg was also examined in a phase II trial in combination with prednisone in the frontline setting. Forty-four patients with a median age of 66 years (range = 26–85 years) were treated, and 41% achieved CMR at 6 months. Median EFS was 14.3 months and median OS was not reached. Notably, cardiovascular adverse events occurred in around 30% of the patients, likely due to the high dose of ponatinib used in this study.^26^

**Table 1.** Low-intensity combinations and chemotherapy-free regimens for Ph-positive ALL.

TKI (year)	*N*	Median age, years (range)	Complete molecular remission	Allogeneic SCT	Relapse-free survival, survival rates/median in months [95% CI^a^]	Overall survival, survival rates/median in months [95% CI^a^]
Low-intensity chemotherapy + TKI						
Imatinib (2015)^27^	135	49 (18–59)	28%	62%	37% (5 years) 30	46% (5 years) 49.2
Dasatinib (2016)^20^	71	69 (59–83)	24%	10%	28% (5 years) 19.1	36% (5 years) 25.8
Dasatinib (2021)^28^	60	42 (19–60)	18%^b^	47%	47% (5 years) 30.4	56% (5 years) Not reached
Nilotinib (2018)^25^	79	65 (55–85)	58%	33%	42% (4 years) –	47% (4 years) –
Nilotinib (2021)^12^	156	47 (18–60)	–	58%	57% (3 years) Not reached	74% (3 years) Not reached
Steroids + TKI						
Imatinib (2007)^29^	29	69 (61–83)	4%	–	48% (1 year) 8 [4–27]	74% (1 year) 20 [12 NE]
Dasatinib (2011)^30^	53	54 (24–77)	23%	34%	51% (1.7 years) 21.5	69% (1.7 years) 30.8
Ponatinib (2022)^31^	44	68 (26–85)	41%	14%	32% (2 years) 14.3 [9.3–22.3]	66% (2 years) Not reached [25.8-NE]
Blinatumomab + TKI						
Dasatinib (2022)^32^	63	54 (24–82)	60%	46%	75% (4 years) Not reached	78% (4 years) Not reached
Ponatinib (2022)^33^	40	57 (20–83)	87%	3%	95% (1 year) Not reached	95% (1 year) Not reached

ALL, acute lymphoblastic leukemia; CI, confidence interval; MRD, measurable residual disease; NE, not estimable; Ph, Philadelphia chromosome; SCT, stem cell transplantation; TKI, tyrosine kinase inhibitor.

a 95% CI: 95% confidence interval included when available.

b The complete molecular response rate was reported at the end of the induction phase with dasatinib plus steroids. The remaining 82% of the patients with MRD positivity received subsequent chemotherapy and allogeneic SCT.

Lower-intensity regimens have also been explored in younger, more fit patients with newly diagnosed Ph-positive ALL. In a randomized study of younger patients with Ph-positive ALL, imatinib was administered in combination with reduced-intensity chemotherapy in 135 patients (median age = 49 years) or Hyper-CVAD in 133 patients (median age = 45 years). The combination of imatinib with reduced-intensity chemotherapy resulted in higher CR rates (98% *versus* 91%; *p* = 0.006) and less induction deaths compared with Hyper-CVAD plus imatinib. This lower-intensity regimen resulted in a 5-year OS of 48%, comparable to the 5-year OS of 43% seen with Hyper-CVAD and imatinib (*p* = 0.37).^27^ This trial suggested that intensive regimens may not be required even for younger, fit patients with Ph-positive ALL. Similarly, in the GIMEMA LAL1509 trial, 60 patients with a median age of 42 years (range = 19–59 years) received induction therapy with steroids given for 31 days plus dasatinib given for 12 weeks.^28^ Patients who achieved CMR after induction were maintained on single-agent dasatinib, while those with residual MRD received consolidation chemotherapy (clofarabine and cyclophosphamide) and allogeneic SCT. At the end of induction therapy, only 11 patients (18%) achieved CMR with this combination, suggesting that an approach of steroids plus a TKI is likely not sufficient for most patients. Among 47 patients with persistent MRD, 22 (47%) underwent allogeneic SCT, of whom 4 relapsed. The cumulative incidence of hematologic relapse, the disease-free survival (DFS), and the OS were all better in patients with CMR on day 85 compared with those with persistent MRD. The differences, however, were not statistically significant probably due to the number of patients and the efficacy of consolidation treatments in inducing MRD negativity. The 5-year DFS and OS rates with this strategy were 47% and 56%, respectively.^28^ This highlights the importance of achieving early MRD negativity for better long-term outcomes and shows that risk-adapted approaches that defer allogeneic SCT in patients with CMR after dasatinib plus steroids are feasible.

Findings from these studies suggest that low-intensity therapies are safe and feasible in patients with newly diagnosed Ph-positive, particularly those who are older and unfit for intensive chemotherapy or allogeneic SCT. Lower-intensity therapies (with low-intensity chemotherapy or steroids in combination with a TKI), however, generally result in inferior long-term survival compared with intensive chemotherapy combinations. The worse survival observed in many of these studies is likely largely due to the lower rates of CMR achieved with lower-intensity therapies, as achievement of a CMR is one of the most important factors determining long-term outcomes in Ph-positive ALL.^28,34^

### Combinations of TKIs with blinatumomab

Blinatumomab is effective as a single agent in relapsed/refractory Ph-positive ALL, with an overall response rate of 36%, a CMR rate of 88% among responders, and a median OS of 9 months.^4,35^ Our group was the first to demonstrate that the combination of blinatumomab and a TKI is highly effective in patients with relapsed/refractory Ph-positive ALL, with a CMR rate of 75% and a 1-year OS of 73%.^5^ Similar outcomes have since been reported by other groups, confirming the feasibility of this chemotherapy-free approach.^6,7^

Encouraging results from these retrospective studies paved the way for further de-escalation strategies and prompted the development of chemotherapy-free regimens with blinatumomab and TKI combinations in Ph-positive ALL. The D-ALBA trial evaluated the combination of dasatinib given for 3 months followed by sequential blinatumomab for up to five cycles.^36^ Sixty-three patients were treated, with a median age of 54 years (range = 24–82 years). Molecular response was achieved in 60% of the patients after two cycles of blinatumomab. Fifteen patients developed rising MRD during dasatinib monotherapy, and six of these patients (40%) were found to have a T315I mutation. Importantly, MRD was eradicated after the addition of blinatumomab in most of these patients, highlighting the efficacy of blinatumomab in this population. After a median follow-up of 40 months, 29 patients (50%) underwent allogeneic SCT in first remission, and the estimated 4-year OS and DFS rates were 78% and 75%, respectively. Nine relapses were observed as follows: four hematologic relapses, four CNS relapses, and one nodal relapse.^32^ This study prospectively confirmed the safety and efficacy of chemotherapy-free regimens in Ph-positive ALL. Notably, however, 50% of patients still underwent SCT, and therefore, the ability of this regimen to overcome the need for SCT in these patients remains uncertain.

Blinatumomab has also been evaluated in combination with ponatinib in a phase II trial among 40 patients with newly diagnosed Ph-positive ALL, with a median age of 57 years (range = 20–83 years).^33^ In contrast with the D-ALBA trial, blinatumomab plus ponatinib were started concomitantly beginning in cycle 1. The CR/CRi rate was 96%, and the overall CMR rates were 87%. After a median follow-up of 15 months, the estimated 1-year EFS and OS rates were both 95%. Importantly, only one patient (3%) underwent allogeneic SCT in first remission (due to persistently detectable *BCR::ABL1* transcript levels). No relapses and no leukemia-related deaths were observed. These findings further highlight a paradigm shift in the management of Ph-positive ALL in which chemotherapy-free regimens are being used, sparing the toxicities of more intensive chemotherapy and mitigating the need for allogeneic SCT.

## CNS prophylaxis

Whether intensive chemotherapy regimens or chemotherapy-free combinations are used, all patients with Ph-positive ALL should receive CNS prophylaxis with intrathecal chemotherapy (IT) to decrease the risk of CNS relapses, which has historically observed in up to 10–15% of patients.^14,37^ Compared with 8 ITs, a total of 12 ITs are associated with higher rates of 3-year (100% *versus* 89%; *p* = 0.049) and 6-year CNS RFS (100% *versus* 91%; *p* = 0.040) and represent the current standard approach for patients treated with intensive chemotherapy–based regimens.^38^ Whether more doses of IT chemotherapy may be needed with the use of chemotherapy-free regimens is unknown. Notably, four of nine relapses in the D-ALBA study were in the CNS, suggesting that more aggressive IT prophylaxis might be needed when these regimens are used.

## Evolving role of allogeneic SCT in Ph-positive ALL

Allogeneic SCT has been historically considered the standard of care for the management of Ph-positive ALL. Newer therapies, however, are re-defining the role of allogeneic SCT in patients with Ph-positive ALL in first remission. The achievement of CMR is established to result in improved outcomes and may be used to identify patients who may not require SCT in first remission. For example, in a study of 85 patients with newly diagnosed Ph-positive ALL treated with Hyper-CVAD plus TKIs without allogeneic SCT, those who achieved CMR within 3 months had superior survival.^34^ The 4-year OS rate for these patients was 66%, and by multivariate analysis, 3-month CMR was the only variable that was prognostic for OS [hazard ratio (HR) = 0.42; *p* = 0.01].^34^ In a follow-up study of 84 patients with Ph-positive ALL treated with frontline Hyper-CVAD plus TKIs who achieved CMR at 3 months, the 5-year progression-free survival (PFS) and OS rates for this population were 68% and 72%, respectively.^39^ By multivariate analysis, ponatinib therapy was factor that independently predicted for relapse (HR = 0.39; *p* = 0.028) and OS (HR = 0.38; *p* = 0.042), while allogeneic SCT in first remission was not prognostic for survival.

These findings suggest that patients with Ph-positive ALL treated with first-line chemotherapy plus TKI, and who achieve early CMR, have an excellent survival and may not need allogeneic SCT. More recently, the combination of blinatumomab plus ponatinib showed encouraging results in the frontline setting, with a CMR rate of 87% and a 1-year OS of 95%, with only 1 of 35 patients (3%) receiving allogeneic SCT.^33^ Thus, this chemotherapy-free combination may eliminate the need for both intensive chemotherapy and the need for allogeneic SCT in the frontline setting. Longer-term follow-up will be needed to confirm the durability of these responses in the absence of SCT in first remission.

## Conclusion

Major progress has been achieved in the management of Ph-positive ALL. The combination of TKIs with intensive chemotherapy improved long-term survival in the frontline setting to up to 75%. More recently, chemotherapy-free regimens with blinatumomab and TKIs have revolutionized the treatment landscape of newly diagnosed Ph-positive ALL. Dasatinib plus blinatumomab yielded similar long-term survival to that achieved with intensive chemotherapy–based regimens. In particular, the combination of ponatinib plus blinatumomab is associated with deep and durable remissions while avoiding cytotoxic therapies and mitigating the need for allogeneic SCT.

In the future, treatment discontinuation may also be feasible in patients with Ph-positive ALL. Currently, patients who do not undergo allogeneic SCT in first remission are often recommended to receive indefinite TKI therapy. Prospective studies are needed to identify patients who can safely discontinue their TKI, similar to what has been seen in chronic myeloid leukemia in which treatment discontinuation is most successful in patients with 5+ years in deep molecular remission.^40–42^ Early achievement of CMR (e.g. within the first cycle of therapy) and more sensitive next-generation sequencing-based MRD assays (sensitivity of) may help to select those who could potentially safely discontinue TKI.^43,44^ Identification of patients who may safely discontinue TKI therapy is important because of the potential for long-term toxicity with all BCR::ABL1 TKIs.

In summary, the management of Ph-positive ALL has witnessed a therapeutic revolution over the past two decades. The incorporation of second- and third-generation TKIs into chemotherapy-free regimens with blinatumomab has transformed the disease from a deadly leukemia to a largely curable one, while sparing the need for cytotoxic chemotherapy and allogeneic SCT in most patients.

## Declarations

**Ethics approval and consent to participate:** Not applicable.

**Consent for publication:** Not applicable.

**Availability of data and materials:** Not applicable.
